# Supplementary material for: MS-222 and Propofol Sedation during and after the Simulated Transport of Nile tilapia (Oreochromis niloticus)
Source: Biology (Basel). 2021 Dec 10;10(12):1309. doi: 10.3390/biology10121309 (PMC8698739; doi:10.3390/biology10121309)
Supplement: Supplementary file 1 [file biology-10-01309-s001.zip › biology-1487515-supplementary.pdf]

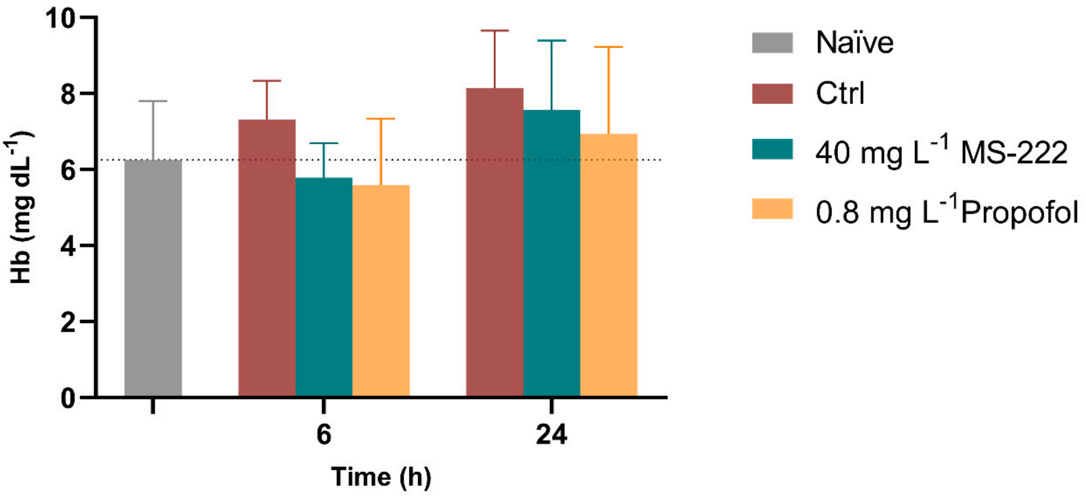

Figure S1 – Haemoglobin (Hb) concentration in blood from Nile tilapia transported in the different experimental conditions for 6 h and after a 24 h recovery period. Data are presented as mean±SD. No statistical differences were observed between experimental groups at any of the analysed time-points ($p>0.05$).

Table S1 – Frequency per min (#) and duration in sec (time) of behaviours analysed during 5 min at the recovery period, 24 h after the simulated transport.

	S		BS		I		EM		IO	MS	C
	#	s	#	time	#	time	#	time	#	#	#
6.5 h											
Ctrl	1.3 (1.2-3.7)	190 (173-305)	0.3 (0.2-0.7)	19 (3-42)	0.7 (0.0-1.3)	46 (0-181)	0.7 (0.2-1.5)	25 (2-154)	0.0 (0.0-0.0)[a]	2.0 (0.0-6.8)	6.5 (1.8-12.5)
MS-222	2.7 (1.0-5.0)	183 (98-311)	0.8 (0.3-1.7)	26 (6-135)	0.8 (0.2-1.2)	79 (14-262)	0.3 (0.0-2.8)	3 (0-105)	0.0 (0.0-0.2)[ab]	1.2 (0.0-4.0)	8.7 (3.2-16.8)
Propofol	2.5 (1.5-4.9)	207 (165-305)	1.8 (0.0-3.2)	154 (0-244)	0.8 (0.0-2.2)	74 (0-184)	0.8 (0.5-2.2)	21 (11-153)	0.2 (0.0-2.7)[b]	1.0 (0.0-1.4)	9.8 (1.2-21.5)
24 h											
Ctrl	2.5 (1.5-3.5)	276 (197-333)	0.0 (0.0-0.5)	0 (0-100)	0.6 (0.0-2.3)	16 (0-137)	1.5 (0.2-2.8)	38 (6-122)	0.0 (0.0-1.3)	2.3 (0.3-2.7)	9.7 (7.7-22.5)
MS-222	2.2 (1.3-5.2)	231 (105-306)	0.0 (0.0-0.7)	0 (0-30)	1.2 (0.2-1.5)	59 (6-256)	1.5 (0.0-3.2)	48 (0-185)	0.0 (0.0-0.3)	1.7 (0.3-5.8)	10.5 (3.3-16.5)
Propofol	3.1 (2.2-3.5)	257 (235-315)	0.2 (0.0-0.7)	5 (0-19)	1.4 (0.8-1.8)	72 (22-86)	1.7 (0.8-2.2)	31 (14-76)	0.6 (0.0-2.5)	1.8 (1.3-2.5)	11.4 (9.5-11.4)

Swimming (S), Bottom swimming (BS), Inactive (I), Erratic movements (EM), Interaction with Objects (IO), Mirror Stimulation (MS) and Crossings (C) obtained from 7 independent replicates and expressed median and interquartile ranges. Statistical analyses were performed using the Kruskal–Wallis test followed by Dunn's multiple-comparison post-hoc tests analysis while the effect of time was tested by Friedmann. Different lowercase letters indicate significant differences between groups ($p<0.05$).

Table S2 – Effects of the different treatments on the pH, redox potential (Eh), colour, drip loss and glucose-based glycogen content at the end of the transportation (6 h) and at the end of the recovery period (24 h).

	pH	Eh (mV)	L*	a*	b*	Chroma	Hue	ΔE[1]	Drip loss (after 7 days)	Glucose-based glycogen (mmol g^{-1} wt)
6 h										
Naïve	6.92±0.19	-50.83±32.02	49.27±2.63	-0.86±0.97	5.78±1.48	5.95±1.32	178.03±4.06	0	3.57±1.07	4.36±1.13
Ctrl	7.01±0.20	-58.86±21.17	48.55±3.30	-1.07±1.09	5.93±2.38	6.13±2.32	180.68±6.49	1.80±1.21	3.94±0.44	4.12±1.39
MS-222	7.14±0.10[A]	-57.71±39.98	49.31±1.66	-1.09±1.03	5.36±2.06	5.64±1.84	171.67±12.3	2.31±1.11	4.32±0.79	4.42±1.97
Propofol	7.10±0.08[A]	-69.57±24.67	49.41±1.27	-2.01±1.41	5.25±1.93	5.69±2.23	180.65±1.07	2.23±1.60	3.25±1.45	3.94±0.36
24 h										
Naïve	6.91±0.09	-61.50±27.33	50.11±1.06	-1.19±0.24	4.49±1.12	4.67±1.04	178.96±2.73	0	3.71±0.56	5.24±1.02
Ctrl	6.82±0.11	-38.50±36.62	48.89±1.73	-1.35±0.42	4.83±1.24	5.04±1.19	180.66±1.34	2.16±1.08	4.22±0.76	4.37±0.50
MS-222	6.75±0.08[B]	-41.17±27.29	47.94±2.02	-0.69±0.43	5.19±1.35	5.26±1.33	179.23±2.56	2.52±1.52	3.92±0.46	4.42±0.63
Propofol	6.81±0.12[B]	-42.17±27.75	48.90±1.65	-1.04±0.46	5.32±1.43	5.46±1.34	181.65±4.45	1.645±0.42	3.86±1.18	5.37±1.76

L* - lightness; a* - redness; b* - yellowness. [1] Total colour difference was normalized to the Naïve group. Data obtained from 7 independent replicates and expressed as mean±SD. Statistical analysis was performed using two-way ANOVA followed by Tukey's multiple-comparison test. Different capital letters represent statistical differences between time within the same group ($p<0.05$).

Table S3 – Biochemical parameters evaluated in different organs at the end of the simulated transportation (6 h) and after the recovery period (24 h).

			ROS (mmol DCF mg protein^{-1})	SOD (KU mg protein^{-1})	CAT (U mg protein^{-1})	GPx (µmol NADPH min^{-1} mg protein^{-1})	GR (µmol NADPH min^{-1} mg protein^{-1})	GST (µmol CDNB min^{-1} mg protein^{-1})	GSH (µmol mg protein^{-1})	GSSG (µmol mg protein^{-1})	OSI	LPO (µmol MDA mg protein^{-1})
Gills	6 h											
		Naïve	0.84 (0.38-1.13)^{ab}	0.98±0.54	0.08 (0.05-0.19)	0.10 (0.06-0.13)	0.05 (0.06-0.09)	0.10 (0.07-0.17)^{ab}	0.03 (0.02-0.04)	0.07±0.06	0.25 (0.17-0.27)	10.3 (2.6-14.2)
		Ctrl	1.05 (0.80-1.47)^{a}	1.36±0.50	0.19 (0.09-0.27)	0.09 (0.04-0.11)	0.15 (0.07-0.20)	0.20 (0.17-0.32)^{a}	0.03 (0.01-0.04)	0.24±0.15	0.11 (0.05-0.24)	12.5 (7.9-19.2)
		MS-222	0.72 (0.51-0.97)^{ab}	0.88±0.31	0.26 (0.18-0.38)	0.05 (0.04-0.11)	0.07 (0.04-0.10)	0.14 (0.10-0.19)^{ab}	0.06 (0.04-0.08)	0.35±0.18	0.14 (0.10-0.19)	16.3 (8.5-22.8)
		Propofol	0.53 (0.32-0.63)^{b}	0.99±0.41	0.11 (0.07-0.21)^{A}	0.07 (0.03-0.11)	0.05 (0.04-0.08)	0.11 (0.09-0.15)^{b}	0.06 (0.04-0.08)	0.23±0.22	0.11 (0.09-0.25)	11.0 (6.0-18.3)
	24 h											
		Naïve	0.72 (0.24-1.09)	0.93±0.38	0.37 (0.24-0.42)	0.05 (0.04-0.13)	0.09 (0.06-0.12)	0.12 (0.09-0.14)	0.04 (0.03-0.07)	0.22±0.08	0.17 (0.10-0.69)	16.1 (11.8-18.6)
		Ctrl	0.89 (0.54-1.02)	1.08±0.34	0.17 (0.09-0.43)	0.07 (0.05-0.14)	0.08 (0.04-0.12)	0.10 (0.08-0.18)	0.02 (0.01-0.03)	0.26±0.23	0.10 (0.07-0.88)	11.8 (5.4-18.1)
		MS-222	0.65 (0.50-0.84)	0.92±0.46	0.19 (0.16-0.50)	0.06 (0.03-0.10)	0.07 (0.03-0.11)	0.10 (0.07-0.17)	0.02 (0.02-0.03)	0.26±0.16	0.23 (0.13-0.25)	11.6 (7.5-15.9)
		Propofol	0.60 (0.39-1.17)	0.93±0.38	0.29 (0.24-0.39)^{B}	0.05 (0.04-0.12)	0.07 (0.04-0.09)	0.10 (0.08-0.13)	0.04 (0.02-0.09)	0.15±0.10	0.44 (0.17-0.59)	15.8 (7.3-20.9)
Muscle	6 h											
		Naïve	0.43±0.17	0.39±0.13	0.37 (0.07-0.59)	0.01 (0.01-0.02)	0.03±0.01	0.03 (0.01-0.03)	0.56±0.28	0.59±0.23	1.16±0.40	15.3±4.90
		Ctrl	0.47±0.25	0.27±0.08	0.43 (0.30-0.79)	0.01 (0.01-0.01)	0.02±0.01	0.02 (0.01-0.03)	0.75±0.28^{A}	0.67±0.32	1.23±0.56^{A}	20.1±9.60
		MS-222	0.44±0.25	0.38±0.05	0.33 (0.23-0.43)	0.02 (0.02-0.03)	0.03±0.01	0.02 (0.01-0.04)	0.51±0.26	0.74±0.49	0.65±0.19	16.6±11.5
		Propofol	0.33±0.16	0.39±0.14	0.25 (0.10-0.64)	0.02 (0.01-0.03)	0.02±0.02	0.02 (0.02-0.04)	0.43±0.31	0.77±0.70	0.66±0.36	15.7±11.3
	24 h											
		Naïve	0.37±0.15	0.33±0.13	0.37 (0.27-0.52)	0.02 (0.02-0.04)	0.03±0.02	0.02 (0.02-0.03)	0.39±0.23	0.59±0.25	0.80±0.25	23.2±18.0
		Ctrl	0.36±0.12	0.34±0.11	0.23 (0.13-0.54)	0.02 (0.01-0.03)	0.03±0.02	0.02 (0.01-0.03)	0.34±0.23^{B}	0.84±0.35	0.46±0.26^{B}	18.1±8.70
		MS-222	0.37±0.14	0.32±0.10	0.16 (0.15-0.63)	0.02 (0.01-0.03)	0.03±0.01	0.02 (0.02-0.03)	0.38±0.15	0.55±0.26	0.89±0.47	20.4±17.0
		Propofol	0.34±0.10	0.28±0.06	0.40 (0.30-0.53)	0.02 (0.02-0.02)	0.02±0.02	0.01 (0.00-0.02)	0.40±0.25	0.74±0.47	0.70±0.35	22.9±13.1
Brain	*6 h*											
		Naïve	0.89±0.52	1.18±0.76	0.07 (0.04-0.22)	0.06±0.02	0.07±0.04^{A}	0.72±0.26	0.07±0.03	0.22±0.14^{ab}	0.23±0.11	2.49±2.49
		Ctrl	0.49±0.23	1.00±0.62	0.11 (0.08-0.22)	0.05±0.02	0.10±0.07	0.38±0.27	0.07±0.08	0.44±0.17^{a}	0.34±0.22	4.83±3.11
		MS-222	0.80±0.22	1.45±0.41	0.24 (0.03-0.26)	0.07±0.02	0.09±0.04	0.65±0.20	0.07±0.04	0.27±0.17^{ab}	0.14±0.07	3.70±3.09
		Propofol	0.77±0.51	1.23±0.74	0.20 (0.09-0.22)	0.05±0.02	0.08±0.03	0.46±0.31	0.07±0.04	0.13±0.07^{b,A}	0.22±0.17	3.66±3.52
	24 h											
		Naïve	0.70±0.36	1.33±0.76	0.04 (0.03-0.14)	0.06±0.04	0.15±0.04^{B}	0.54±0.22	0.06±0.06	0.14±0.11	0.45±0.29	4.14±2.32
		Ctrl	0.59±0.31	1.19±0.76	0.12 (0.06-0.27)	0.08±0.05	0.10±0.08	0.52±0.27	0.07±0.05	0.30±0.20	0.22±0.14	5.02±3.93
		MS-222	0.67±0.18	1.55±0.80	0.21 (0.08-0.32)	0.04±0.04	0.10±0.04	0.63±0.18	0.06±0.05	0.29±0.24	0.19±0.10	4.82±2.68
		Propofol	0.80±0.41	0.64±0.34	0.19 (0.14-0.34)	0.06±0.04	0.09±0.05	0.53±0.26	0.13±0.09	0.38±0.11^{B}	0.24±0.20	4.55±3.05
Liver	*6 h*											
		Naïve	1.52 (1.22-1.70)	0.53 (0.37-0.62)	2.01 (1.41-2.52)^{A}	0.04±0.01	0.02 (0.02-0.03)^{a,A}	0.27±0.11	0.31 (0.16-0.41)	4.71±0.23	0.07±0.03	50.1 (33.0-68.9)
		Ctrl	1.20 (0.49-1.32)	0.80 (0.58-0.85)	0.86 (0.59-1.36)	0.05±0.02	0.05 (0.03-0.08)^{b,A}	0.25±0.15	0.22 (0.20-0.37)	4.33±3.16	0.09±0.05	51.1 (20.2-59.3)
		MS-222	1.10 (0.77-1.37)	0.67 (0.59-0.68)	1.01 (0.66-1.44)	0.05±0.02	0.03 (0.02-0.05)^{ab}	0.27±0.14	0.22 (0.12-0.37)	4.32±2.53	0.07±0.04	22.8 (17.9-42.4)
		Propofol	0.98 (0.67-1.32)	0.58 (0.48-0.69)	1.31 (1.11-1.56)	0.05±0.01	0.04 (0.02-0.08)^{ab}	0.30±0.15	0.28 (0.18-0.50)	4.46±3.03	0.09±0.03	33.6 (28.6-35.8)
	24 h											
		Naïve	0.99 (0.54-1.49)	0.49 (0.41-0.63)	1.04 (0.65-1.49)^{B}	0.04±0.01	0.08 (0.04-0.09)^{a,B}	0.31±0.24	0.22 (0.11-0.46)	4.40±2.93	0.07±0.04	65.7 (36.1-74.8)
		Ctrl	1.07 (0.95-1.78)	0.47 (0.38-0.70)	1.23 (0.49-1.39)	0.04±0.02	0.02 (0.01-0.03)^{ab,B}	0.34±0.10	0.31 (0.15-0.41)	3.68±0.98	0.09±0.04	33.4 (24.9-50.0)
		MS-222	1.06 (0.81-1.30)	0.51 (0.45-0.70)	1.09 (0.71-1.20)	0.04±0.02	0.02 (0.02-0.05)^{ab}	0.28±0.14	0.25 (0.13-0.43)	4.67±2.77	0.08±0.06	51.1 (24.7-56.9)
		Propofol	0.93 (0.73-1.27)	0.47 (0.36-0.58)	0.85 (0.38-1.70)	0.04±0.01	0.01 (0.01-0.02)^{b}	0.31±0.11	0.24 (0.11-0.38)	4.48±3.16	0.07±0.03	48.8 (31.1-67.9)

Data from 7 independent replicates expressed as mean ± SD for parametric data distribution or median and interquartile range for non-parametric data. Statistical analysis was performed using one-way ANOVA followed by Tukey's multiple-comparison test or Kruskal-Wallis followed by Dunn's multiple-comparison post-hoc tests. Different lowercase letters indicate significant differences between groups while capital letters represent statistical differences between time within the same group ($p<0.05$).
